# Comparing the effectiveness of the BPMAP (Blood Pressure Management Application) and usual care in self-management of primary hypertension and adherence to treatment in patients aged 30–60 years: study protocol for a randomized controlled trial

**DOI:** 10.1186/s13063-016-1638-0

**Published:** 2016-10-21

**Authors:** Mahnaz Ashoorkhani, Ali Bozorgi, Reza Majdzadeh, Hamed Hosseini, Ali Yoonessi, Ali Ramezankhani, Hassan Eftekhar

**Affiliations:** 1 Department of Health Education and Promotion, School of Public Health, Tehran University of Medical Sciences, Tehran, Iran; 2 Tehran Heart Center, Tehran University of Medical Sciences, Tehran, Iran; 3 School of Public Health, Knowledge Utilization Research Center, Tehran University of Medical Sciences, Tehran, Iran; 4 Clinical Trial Center, Tehran University of Medical Sciences, Tehran, Iran; 5 Department of Neuroscience, School of Advanced Technologies in Medicine, Tehran University of Medical Sciences, Tehran, Iran; 6 Department of Public Health, Shahid Beheshti University of Medical Sciences, Tehran, Iran

**Keywords:** Hypertension, m-Health, Self-management, Adherence to treatment

## Abstract

**Background:**

Hypertension is one of the most important and well-known risk factors for cardiovascular diseases. Unfortunately, in spite of effective treatments, adherence to the regular use of drugs and other nondrug treatments, such as lifestyle improvement, is often poor. This study evaluates the effectiveness of an educational, supportive intervention – in the form of a Blood Pressure Management Application (BPMAP) – on self-management in patients with primary hypertension on controlling the determinant factors of hypertension, and on adherence to treatment.

**Methods/design:**

A two-arm, parallel-design randomized controlled clinical trial will be conducted on 30 to 60 year-old patients with primary hypertension who are attending the Tehran Heart Center. One hundred and thirty-two (132) patients will be randomly assigned to the intervention and control (usual method) groups. The most important inclusion criteria are, having primary hypertension and being pharmacologically treated for it, and not having developed the complications of hypertension, such as myocardial infarction, cerebral stroke and cardiac insufficiency. The participants should be able to read Persian and be able to use the application.

The most important outcomes of the study include adherence to treatment, weight control, and regular monitoring of blood pressure which are assessed in the primary assessment (baseline data questionnaire) and again at the 8^th^ and 24^th^ weeks. The intervention is a mobile application that has capabilities such as reminders and scientific and supportive information.

**Discussion:**

This application has been programmed to reduce many of the nonadherence factors of hypertension treatment. Therefore, the findings may contribute to a rise in adherence to treatment. If proven to have an appropriate impact, it may be extended for use in the national hypertension control plan.

**Trial registration:**

This study was registered in the Iran Randomized Clinical Trial Center under the number IRCT2015111712211N2 on 1 January 2016.

**Electronic supplementary material:**

The online version of this article (doi:10.1186/s13063-016-1638-0) contains supplementary material, which is available to authorized users.

## Background

Cardiovascular diseases (CVD) are the primary worldwide cause of morbidity and mortality. The death rate caused by CVD is expected to reach 23.6 million by 2030 [1]. Hypertension (HTN) is second only to obesity in its contribution to the etiology of CVD. With every 10 mmHg elevation in blood pressure (BP), the risk of CVD increases by 30 % [2]. Adherence to drugs, weight loss, smoking cessation, the Dietary Approaches to Stop Hypertension (DASH) diet plan and a low-salt diet, and taking appropriate exercise – particularly aerobic – are among the most important factors of disease control [2]. In spite of effective medical treatments for HTN, patients’ level of adherence to regular drug use has been reported at only 30–50 % at the international level [3].

Some of the reasons for nonadherence to treatment are: the drugs’ side effects; the patient’s disbelief in the efficacy and benefit of therapy; lack of motivation; the absence of bothersome physical symptoms in some patients; lack of knowledge and appropriate behavior toward drug therapy and dietary regimens; miscommunication between the patient and physician; the complexity of therapy; inadequate attendance for follow-up on the part of the patient, and certain psychological issues such as depression [2, 4].

The widespread use of smart phones enables their use – in the form of mobile applications – for beneficial medical and health-related purposes [5]. Nowadays, clinical applications can be used in smart phones as they have the following capabilities; audio-visual communication, Short Message Service (SMS), multimedia, medical sensors, the ability to connect to various devices for registering vital signs, such as electrocardiographic (ECG) monitoring, BP monitoring, the automatic transfer of data to registry systems, and the ability to connect to the Internet [6, 7]. Many studies have examined the effectiveness of mobile applications, most of which have reported positive results [1, 7].

As mentioned earlier, adherence to treatment is not favorable in spite of effective therapies. Here, we intend to evaluate the impact of an intervention aimed at overcoming the many barriers of adherence in HTN. The intervention has been designed in the form of the Blood Pressure Management Application (BPMAP) software application to enhance self-management in the control of the disease and to improve adherence in patients with primary HTN.

## Methods/design

### Overview

The main goal of this study is to evaluate the effect of the BPMAP mobile application on self-management in hypertensive patients. The application has been programmed to enhance individuals’ knowledge of the disease, its subsequent problems and methods of control, regular use of drugs, observance of the low-sodium and DASH diets, level of physical activity, smoking cessation, and weight loss. The assumption here is that this application can enable individuals to better control the determinant factors of HTN and reduce the possibility of its complications.

### Study design

This study is a randomized controlled, parallel-design clinical trial intended to demonstrate the impact of 6 months’ use of the BPMAP application on self-management in hypertensive patients, on their adherence to treatment, and the control of their risk factors for HTN.

The protocol is guided by the Standard Protocol Items: Recommendations for Interventional Trials (SPIRIT) 2013 Statement (see Additional file 1).

### Study setting

The participants will be selected from the patients attending the Tehran Heart Center Clinic. The Tehran Heart Center is a tertiary-care, educational subspecialty center for CVD affiliated with Tehran University of Medical Sciences. It has 460 beds and is one of the most well- equipped, specialized care centers for CVD in the region. Annually, an average 113,316 outpatients, 23,879 inpatients and 4331 open-heart surgery candidates are treated at the center [8].

**Fig. 1:**
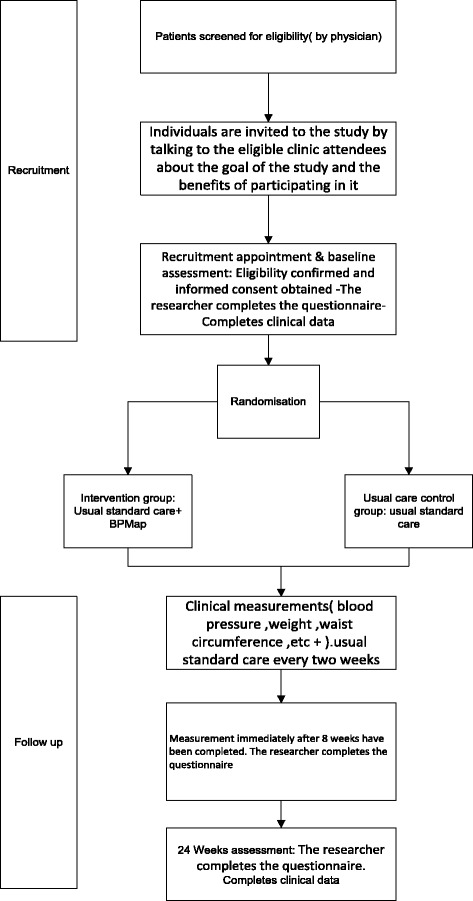
The procedure of the study evaluating the impact of the Blood Pressure Management Application (BPMAP)

The outcomes of the study (adherence to treatment, weight loss, regular BP monitoring) will be evaluated at the primary assessment (baseline data questionnaire) and again at the 8^th^ and 24^th^ weeks (Fig. 1).

## Intervention

### Intervention and control groups

In addition to the usual care, the participants of the intervention group will receive the educational, supportive mobile application-based intervention (Additional file 2). The participants will have these applications for 6 months and the aforementioned measurements will be registered in it. Reminders for dose and time of drug intake, date of clinical visit, and other reminders will be activated. The users will be provided with the scientific information and skills required to identify the disease and its methods of control by the mobile application.

The participants in the comparison arm will receive the usual standard care based on the Joint National Committee (JNC) guideline. The usual care includes, measuring the patient’s BP, necessary assessments based on the patient’s history and circumstances, the specialist physician’s judgment, medication prescription, and other medical measures. The number of appointments and assessments in each group will be the same. The intervention and control participants’ appointment days will differ and the members of one group will not be in touch with those of the other.

The BPMAP application contains educational content that is meant to raise the level of knowledge in hypertensive patients regarding its complications and methods of control. Moreover, it contains supportive measures such as important reminders for time of drug use, smoking cessation, and weight loss.

### Intervention delivery

The software will be installed on the mobile phone and its operation will be taught individually. The patients will work with the application in the presence of the research team, and any existing problems will be resolved. Since BP measurement will take place at the individual’s home and with a personal sphygmomanometer, the patients’ personal sphygmomanometers will be calibrated at the same time that the application is installed. In case there is a large difference in readings, or if the sphygmomanometer is faulty, the patient will be advised to change it. The strategies foreseen for adherence improvement include reminders set at defined intervals in the form of pop-up messages. In order to monitor adherence, the data collected on the server will be used.

In addition to the application, a server has been designed in which the users’ activities will be collected. Items such as the duration the application has been left open by the user, the sections used by the user (in addition to registering their time and duration), and BP registration, etc. will be registered. Every time the user’s mobile is connected to the Internet, the data will be uploaded and saved on to the server. These data can be used as a proxy of adherence to the intervention.

## Outcomes

### Primary outcomes/clinical

The preintervention clinical measurements will be taken at weeks 8 and 24. These measurements include: systolic and diastolic BP, total cholesterol level, high-density lipoprotein (HDL) level, low-density lipoprotein (LDL) level, Body Mass Index (BMI), and waist circumference.

### Primary outcomes/behavioral

Assessment of health behaviors affecting hypertension, such as healthy diet, physical activity level, checking and monitoring BP, adherence to treatment, and predisposing factors, enabling factors and reinforcing factors contributing to adherence to treatment, and smoking cessation.

#### Adherence to antihypertensive medication use

This will be assessed with the Hill-Bone High Blood Pressure Therapy Compliance Scale, which includes 14 items, at the initial and final assessments [9–11]. Individuals will score from 14 to 56. An increase exceeding 20 % of the Compliance Scale score is considered effective.

#### Adherence to the DASH and low-sodium diets

A couple of questions in the baseline and final questionnaires will evaluate this. A 20 % rise in the score in this section is considered desirable.

#### Physical activity

The change and sequence of physical activity will be evaluated through a couple of questions in the questionnaire, at the baseline assessment and at the 8^th^ and 24^th^ weeks.

#### Regular BP monitoring

BP can be regularly monitored by regularly measuring and saving the relevant data in the mobile app. For every timely registration a score will be awarded. This part of the study is 60 days long. The participants will be asked to measure and register their BP once every other day. A score of 25 and higher will be considered appropriate.

#### Smoking cessation

This will be evaluated through a couple of questions in the questionnaire, at the baseline assessment and at the 8^th^ and 24^th^ weeks.

#### Predisposing factors, enabling factors and reinforcing factors of adherence

Some of the questions of the questionnaire assess these factors. These measurements are performed at the baseline assessment and at the 8^th^ and 24^th^ weeks. A 20 % increase in score is considered desirable.

## Outcome measures

The primary outcomes of the intervention will be measured by, the researcher-made questionnaire, a manual sphygmomanometer, a weighing scale, a measuring tape, and biological tests.

### Questionnaire

The questionnaire contains the patient’s personal and demographic information, treatment status, and certain health-related behaviors as the baseline assessment. In creating it, we have used some of the constructs taken from the Predisposing, Reinforcing and Enabling Constructs in Educational Diagnosis and Evaluation (PRECEDE) model (predisposing, enabling and reinforcing factors), the WHO STEPS questionnaire for monitoring of chronic diseases risk factors [12], the Hill-Bone High Blood Pressure Therapy Compliance Scale [9], and the International Physical Activity Questionnaire (IPAQ) [13].

## Participants

The participants are hypertensive patients who have been diagnosed by a physician. The inclusion criteria are, age 30 to 60 years, treatment for HTN in the past year, having a smart phone and the ability to use it, sufficient literacy, inclination to participate in the study, and residence at the study’s location in the first 6 months of the study. The exclusion criteria include having had complications of HTN, such as myocardial infarction, cardiac insufficiency and other cardiovascular diseases, diabetes, and other physical disabilities. After obtaining informed consent from all the participants, their inclusion criteria will be evaluated by a physician. Then, the baseline data will be collected by the abovementioned (baseline data) questionnaire.

### Sample size

The sample size is 120 (60 persons in each group) with a power of 80 % to detect a minimum difference of five scores (a mean of 40 and 45 in the control and intervention groups and a standard deviation of 10). Based on the Hill-Bone High Blood Pressure Therapy Compliance Scale [9], the mean difference was calculated with the two-tailed test at a significance level of 5 %. Bearing in mind the possibility of sample loss, 10 % was added to the sample size and 132 persons were estimated.

### Randomization

The participants will be randomly assigned to two groups after the initial examination and upon completing the baseline data form. Random sequencing will be done in blocks of 4 using the randomization website at http://www.jerrydallal.com/random/permute.htm. The ratio of assignment to each arm will be 1:1 and each group will have 66 individuals.

### Follow-up

The participants will be examined seven times at 2-week intervals (Table 1). In the first visit, after ensuring the participant’s inclination to participate in the study and after the informed consent form is completed, the baseline data questionnaire will be completed by the researcher. Clinical measurements, such as systolic and diastolic BP, waist circumference and hip circumference, BMI, and biochemical measurements, such as LDL, HDL, and total cholesterol levels, will be performed.

**Table 1:** Steps taken in each visit

	Study period										
	Enrollment	Allocation	Post allocation								Close-out
Week	−1 to 0	0 ± 2 days	2 ± 2 days	4 ± 2 days	6 ± 2 days	8 ± 2 days					24 ± 2 days
Time point	1^st^ visit	2^nd^ visit	3^rd^ visit	4^th^ visit	5^th^ visit	6^th^ visit					7^th^ visit
Enrollment:	×										
Eligibility screen	×										
Informed consent	×										
Allocation	×										
Intervention		×	×	×	×	×	×	×	×	×	×
Assessment	×					×					×
Baseline variables	×					×					×
Measuring adherence to treatment	×					×					×
Measuring adherence to the diet	×					×					×
Evaluating the predisposing, enabling, and reinforcing factors of adherence	×					×					×
Measurement of systolic and diastolic BP, waist circumference, and BMI	×	×	×	×	×	×	×	×	×	×	×
Evaluating the regular monitoring of BP by the mobile application	×	×	×	×	×	×	×	×	×	×	×
Evaluating the usability of the software						×					×
Evaluating the level of satisfaction of the software/application						×					×

*BMI* Body Mass Index, *BP* blood pressure

The 2nd visit will be made a week after the first. The application will be installed on the cell phones of the intervention group’s patients and its operation will be taught to them. Any problems that arise will be dealt with accordingly. Moreover, the sphygmomanometer used by the patient at home will be evaluated for its precision and sensitivity (calibration). In order to ensure that the patients measure their BP correctly at home, the methods applied by them will be controlled, and if faulty, corrected. Clinical measurements and usual care will be performed. The usual care includes history taking, BP measurement, and diagnostic and therapeutic measures based on the JNC guideline on high blood pressure.

From the 2nd visit onward (up to the 6th visit) patients will be visited every 2 weeks. In each visit the patient’s BP, weight and waist circumference will be measured, and standard antihypertensive therapeutic measures – based on the JNC guideline – will be taken.

On the 6th visit, in addition to the assessments made throughout the 2^nd^ to 5^th^ visits, the baseline data questionnaire will be completed for the patients as well.

On the 7th visit, which will be conducted 16 weeks after the 6^th^ visit, the baseline data questionnaire as well as clinical assessments will be evaluated.

On the 6th and 7th visits, the intervention group’s patients will be asked whether they are familiar with another person involved in this study. If the response is positive they will be asked further questions and the data will be included in the analysis. This question will be included to take into account the possibility of contact between the participants of the control and intervention groups.

### Withdrawal criteria

The participant will be excluded from the study if any of the following are seen during the study:

1. Lack of registration of data in the application for more than a week
2. Lack of interest in continuing to participate in the study
3. Nonattendance for two consecutive visits
4. Change in dose and type of antihypertensive medication
5. Hospital admission due to CVD
6. Emigration from Tehran
7. Death

### Data management

Patient information will be collected from two sources: (1) digital data collected on the server and (2) a questionnaire assessing risk factors and adherence to treatment, which is on paper and which will be completed by the researcher. The entire data will be prepared from a SPSS database based on a pre-defined data dictionary. To ensure the validity of data, data entry will take place through double entry.

## Statistical methods

The qualitative findings of the study will be described using frequency and percentage frequency, and the quantitative findings will be described using mean and standard deviation. To analyze the effectiveness of the main outcome variable of the study, we will use analysis of covariance (comparison of mean changes in score of the adjusted questionnaire – based on the baseline score at the beginning of the study and other possible confounding factors) at a confidence interval of 95 %. This approach will be repeated for all the quantitative variables.

The remaining qualitative variables of the study will be compared by the chi-squared test between the two groups. The main approach of the study will be intention-to-treat. The final results will be analyzed per protocol. Lost data will be replaced through multiple imputation techniques.

## Discussion

The goal of controlling and treating HTN is to prevent complications such as cardiovascular, renal, cerebrovascular and other diseases. Luckily, there are effective treatments for HTN. The JNC HTN treatment management guideline – compiled approximately every 4 years by the American Heart Association – is the basis of the standard treatment of HTN [3]. In addition to adherence to treatment, hypertensive patients need to self-manage their health behaviors such as weight loss, smoking cessation, eating a low-sodium diet, and appropriate exercise – particularly aerobic. However, in spite of the availability of medication patient adherence to regular drug intake has been reported at only 30–50 % [14]. According to the WHO’s 2002 report, drug compliance in the long-term treatments of chronic diseases overall among developed countries is only 50 % [14]. Any of the following reasons may contribute to this noncompliance: treatment of asymptomatic patients, drug side effects, the patient’s disbelief in the effectiveness and benefit of treatment, lack of motivation, insufficient awareness of the individual of the disease and its physical complications, lack of adequate knowledge and practice on the required drug treatment and nutritional diet, miscommunication between the patient and physician, the complexity of treatment, the cost of treatment, insufficient attendance for follow-up on the part of the patient, and the existence of certain psychological problems such as depression [15].

The emergence of smart phones has made its application in various fields possible, including that of medicine. The use of mobile-based technologies in health-related topics is on the rise owing to their easy application, low cost, accessibility unbound by time and place, and overcoming the issue of distance in access to services [16]. Here we will use the BPMAP application to enable, identify and self-manage and optimally control the determinant factors of HTN and overcome the barriers to adherence to treatment. The content of the program has been designed according to the constructs of a behavior-change model, the PRECEDE model. These constructs include: (1) predisposing factors such as knowledge and attitude, (2) enabling factors, which are those factors that prepare the ground for the behavior change, and (3) reinforcing factors, which are those factors that result in the adoption of a different behavior and a rewarding of persistence of that particular behavior change [17, 18].

Among the limitations of this study is the self-report nature of the questionnaire and the need to register the measurements in the application. The necessity of owning a smart phone is another limitation. Some of the strengths of the study are: (1) using the mobile phone as an intervention tool, a technology that is ubiquitous and has no temporal and spatial limitations, (2) safeguarding individuals’ privacy at the time of data delivery and reception of feedback appropriate to the individuals’ data, (3) sampling of both control and intervention groups from the same medical center, and (4) the low cost of the study.

## Trial status

This trial is at the recruitment stage.

## Additional files

Additional file 1: SPIRIT 2013 checklist: recommended items to address in a clinical trial protocol and related documents*. (DOCX 41 kb)
Additional file 2: The design and properties of the application. (DOCX 15 kb)

## Abbreviations

BMI: Body Mass Index
BP: Blood pressure
BPMAP: Blood Pressure Management Application
CVD: Cardiovascular diseases
DASH: Dietary Approaches to Stop Hypertension
ECQ: Electrocardiogram
HDL: High-density lipoprotein
HTN: Hypertension
IPAQ: International Physical Activity Questionnaire
JNC: Joint National Committee
LDL: Low-density lipoprotein
PRECEDE: Predisposing, Reinforcing and Enabling Constructs in Educational Diagnosis and Evaluation
SMS: Short Message Service
WHO: World Health Organization

## References

[1] Raghu A, Devarsetty P, Peiris D, Clifford G, Tarassenko L (2013). Engineering a mobile health tool for resource-poor settings to assess and manage cardiovascular disease risk: SMARThealth study. BMC Med Inform Decis Mak.

[2] Bolaji A (2014). Simulation of a real-time mobile health monitoring system model for hypertensive patient in rural Nigeria. Afr J Comp ICT.

[3] James PA, Oparil S, Carter BL, Cushman WC, Dennison-Himmelfarb C, Handler J, Lackland DT, LeFevre ML, MacKenzie TD, Ogedegbe O (2014). 2014 evidence-based guideline for the management of high blood pressure in adults: report from the panel members appointed to the Eighth Joint National Committee (JNC 8). JAMA.

[4] Kahana S, Drotar D, Frazier T (2008). Meta-analysis of psychological interventions to promote adherence to treatment in pediatric chronic health conditions. J Pediatr Psychol.

[5] Martínez-Pérez B, de la Torre-Díez I, López-Coronado M, Herreros-González J. Mobile apps in cardiology: review. JMIR mHealth and uHealth. 2013; doi:10.2196/mhealth.2737. 10.2196/mhealth.2737 PMC4114428 25098320

[6] Jones V, Gay V, Leijdekkers P. Body sensor networks for mobile health monitoring: Experience in europe and australia. In Digital Society, 2010 ICDS'10 Fourth International Conference on. IEEE; 2010:204-9.

[7] Honeyman E, Ding H, Varnfield M, Karunanithi M (2014). Mobile health applications in cardiac care. Interv Cardiol.

[8] Tehran Heart Center. 2016. http://thc.tums.ac.ir/En/. Accessed 20 Sept 2016.

[9] Kim MT, Hill MN, Bone LR, Levine DM (2000). Development and testing of the Hill‐Bone Compliance to High Blood Pressure Therapy Scale. Prog Cardiovasc Nurs.

[10] Song Y, Han H-R, Song H-J, Nam S, Nguyen T, Kim MT (2011). Psychometric evaluation of Hill-Bone Medication Adherence Subscale. Asian Nurs Res.

[11] Dehghan M, Nayeri ND, Iranmanesh S (2015). Validating the Persian version of the Hill-Bone’s Scale of “Compliance to High Blood Pressure Therapy.”. Br J Med Med Res.

[12] Instrument WS, The WHO (2008). STEPwise approach to chronic disease risk factor surveillance (STEPS).

[13] Committee IR. Guidelines for data processing and analysis of the International Physical Activity Questionnaire (IPAQ)—short and long forms. Retriev Sept. 2005;17:2008.

[14] Goldstein CM, Gathright EC, Dolansky MA, Gunstad J, Sterns A, Redle JD, Josephson R, Hughes JW (2014). Randomized controlled feasibility trial of two telemedicine medication reminder systems for older adults with heart failure. J Telemed Telecare.

[15] Osterberg L, Blaschke T (2005). Adherence to medication. N Engl J Med.

[16] Rich MW, Beckham V, Wittenberg C, Leven CL, Freedland KE, Carney RM (1995). A multidisciplinary intervention to prevent the readmission of elderly patients with congestive heart failure. N Engl J Med.

[17] Glanz K, Rimer BK, Viswanath K. Health behavior and health education: theory, research, and practice: John Wiley & Sons; 2008.

[18] Green LW, Kreuter MW (2005). Health program planning: an educational and ecological approach.

