# Supplementary material for: Comparing the effectiveness of the BPMAP (Blood Pressure Management Application) and usual care in self-management of primary hypertension and adherence to treatment in patients aged 30–60 years: study protocol for a randomized controlled trial
Source: Trials. 2016 Oct 21;17:511. doi: 10.1186/s13063-016-1638-0 (PMC5073944; doi:10.1186/s13063-016-1638-0)
Supplement: Additional file 2: — The design and properties of the application. (DOCX 15 kb) [file 13063_2016_1638_MOESM2_ESM.docx]

**Additional file 2 – The design and properties of the application**

The contents of the BPMap mobile application were developed based on the needs assessment conducted on hypertensive patients. Needs assessment was conducted qualitatively. In this method, some of the constructs of the PRECEDE – PROCEED model were used for designing the questions. The resultant content was examined by both experts and hypertensive patients and modified according to their opinions. The final content was prepared with the help of application developers in the form of the BPMap software. The educational content will be arranged in the form of a software program through five stages [19].

1-Defining the conceptual framework, bearing in mind the system’s properties and development of appropriate content, 2-Approving the conceptual framework and system of delivery, 3-Arrangement and adjustment of the conceptual framework and content (tailoring), bearing in mind the users’ characteristics, 4-Performance assessment, and, 5-Data collection and analysis. The 4^th^ and 5^th^ stages will be conducted through the controlled randomized clinical trial protocol.

The BPMap contains the following items:

- Reminder for taking medications, the date of the medical visit, physical activity, and blood pressure
- Scientific information on the disease, its etiology, causative factors, factors affecting disease control
- Scientific and encouraging information and reminders for smoking cessation
- Presenting the healthy DASH and low-sodium diet based on the individual’s BMI and other characteristics
- Registration of BP in the software and provision of advice in line with the BP registered in the software
- Teaching the necessary conditions and measurement of BP at home
- Delivery of data to the physician and researcher
- Delivery of an SMS alert and/or notification to a family member’s phone, in case a borderline BP is registered
